# Impact of short-term dietary modification on postprandial oxidative stress

**DOI:** 10.1186/1475-2891-11-16

**Published:** 2012-03-21

**Authors:** Richard J Bloomer, John F Trepanowski, Mohammad M Kabir, Rick J Alleman, Michael E Dessoulavy

**Affiliations:** 1 Cardiorespiratory/Metabolic Laboratory, The University of Memphis, Memphis, TN 38152, USA; 2 School of Physical Education, University of Otago, Dunedin, New Zealand

**Keywords:** Dietary restriction, Oxidative stress, Antioxidants, Postprandial, Reactive oxygen species

## Abstract

**Background:**

We have recently reported that short-term (21-day) dietary modification in accordance with a stringent vegan diet (i.e., a Daniel Fast) lowers blood lipids as well as biomarkers of oxidative stress. However, this work only involved measurements obtained in a fasted state. In the present study, we determined the postprandial response to a high-fat milkshake with regards to blood triglycerides (TAG), biomarkers of oxidative stress, and hemodynamic variables before and following a 21-day Daniel Fast.

**Methods:**

Twenty-two subjects (10 men and 12 women; aged 35 ± 3 years) completed a 21-day Daniel Fast. To induce oxidative stress, a milkshake (fat = 0.8 g·kg^-1^; carbohydrate = 1.0 g·kg^-1^; protein = 0.25 g·kg^-1^) was consumed by subjects on day one and day 22 in a rested and 12-hour fasted state. Before and at 2 and 4 h after consumption of the milkshake, heart rate (HR) and blood pressure were measured. Blood samples were also collected at these times and analyzed for TAG, malondialdehyde (MDA), hydrogen peroxide (H_2_O_2_), advanced oxidation protein products (AOPP), nitrate/nitrite (NOx), and Trolox Equivalent Antioxidant Capacity (TEAC).

**Results:**

A time effect was noted for HR (*p *= 0.006), with values higher at 2 hr post intake of the milkshake as compared to pre intake (*p *< 0.05). Diastolic blood pressure was lower post fast as compared to pre fast (*p *= 0.02), and a trend for lower systolic blood pressure was noted (*p *= 0.07). Time effects were noted for TAG (*p *= 0.001), MDA (*p *< 0.0001), H_2_O_2 _(*p *< 0.0001), AOPP (*p *< 0.0001), and TEAC (*p *< 0.0001); all concentrations were higher at 2 h and 4 h post intake compared to pre intake, except for TEAC, which was lower at these times (*p *< 0.05). A condition effect was noted for NOx (*p *= 0.02), which was higher post fast as compared to pre fast. No pre/post fast × time interactions were noted (*p *> 0.05), with the area under the curve from pre to post fast reduced only slightly for TAG (11%), MDA (11%), H_2_O_2 _(8%), and AOPP (12%), with a 37% increase noted for NOx.

**Conclusion:**

Partaking in a 21-day Daniel Fast does not result in a statistically significant reduction in postprandial oxidative stress. It is possible that a longer time course of adherence to the Daniel Fast eating plan may be needed to observe significant findings.

## Background

Modifying dietary intake in a manner that reduces kilocalorie ingestion and emphasizes the consumption of nutrient dense, plant-derived foods such as fruits, vegetables, whole grains, nuts and seeds [1-3] yields favorable health effects. Such modifications typically increase the intake of antioxidant micronutrients [4,5], thus improving blood antioxidant status [6,7] and possibly decreasing the production of reactive oxygen species (ROS) [8]. Moreover, the restriction in overall dietary energy [9], in addition to a specific decrease in dietary protein [10,11], has been reported to reduce ROS, suggesting that these dietary modifications may collectively attenuate oxidative stress [12]. Finally, increasing the intake of plant-based polyphenols [13] and nitrates [14] may improve endothelial function by increasing nitric oxide bioavailability (which is often verified by measuring the surrogate marker, nitrate/nitrite [NOx]).

One way for individuals consuming a typical Western diet to realize the dietary modifications mentioned in the previous paragraph is to partake in a Daniel Fast. The concept of a Daniel Fast originates from two passages within Biblical text (Daniel 1:8-14 and Daniel 10:2-3). This diet allows for *ad libitum *intake of fruits, vegetables, whole grains, nuts, seeds, legumes, and oil. Thus, the Daniel Fast resembles a vegan diet, which has been reported to yield health-enhancing benefits [15-17]. However, in addition to prohibiting the consumption of animal products, the Daniel Fast also prohibits the consumption of processed foods, white flour products, preservatives, additives, sweeteners, flavorings, caffeine, and alcohol.

We have recently reported that individuals partaking in a 21-day Daniel Fast experience increases in antioxidant capacity and reductions in oxidative stress biomarkers [12] and certain blood lipid concentrations [18] when measured in a fasted state. This suggests that a 21-day Daniel Fast may attenuate postprandial oxidative stress (i.e., oxidative stress that is specifically caused by the metabolism of a consumed meal [19], particularly a meal that is high in saturated fat [20,21]). There are two main reasons for this assertion. First, postprandial oxidative stress appears to be positively correlated with basal oxidative stress and negatively correlated with basal antioxidant capacity [22]. Second, postprandial lipid concentrations - which are strongly correlated with the postprandial increase in ROS production [20-23] - appear to be positively correlated with fasting lipid concentrations (triglycerides in particular).

Although our prior work with the Daniel Fast yielded favorable findings with regards to a wide range of health-related variables (including blood lipids and oxidative stress biomarkers), we only included measurements obtained in a fasted state. Therefore, the purpose of the present study was to determine the postprandial response to a high-fat milkshake meal with regards to biomarkers of oxidative stress (as well as related variables) both before and following a 21-day Daniel Fast. We hypothesized that partaking in a 21-day Daniel Fast would favorably alter the postprandial response to the milkshake meal (defined as a decrease in postprandial blood lipid concentrations and/or a decrease in postprandial oxidative stress biomarker concentrations).

## Methods

### Subjects

Twenty-two subjects (10 men and 12 women) were included. In our initial work with the Daniel Fast, we noted no differences in the changes in our outcome measures between individuals of different body mass index (BMI) [18]. Therefore, we did not place any restrictions on BMI for enrollment in the present study. Subjects' BMI ranged from 18 kg·m^−2 ^to 37 kg·m^−2^, with 13 subjects classified as normal weight (BMI <25 kg·m^−2^), 4 classified as overweight (BMI 25-29.9 kg·m^−2^), and 5 classified as obese (BMI ≥30 kg·m^−2^). Subjects were nonsmokers and were not consuming antioxidant supplements from 2 weeks prior to starting the fast until the conclusion of the fast. Nineteen of the 22 subjects were classified as exercise-trained, performing 2.1 ± 0.3 h per week of anaerobic exercise for the past 7.7 ± 1.4 years and 4.3 ± 0.4 h per week of aerobic exercise for the past 8.6 ± 1.2 years.

### Initial lab visit

Eligibility and classification were determined via completion of questionnaires pertaining to health history, physical activity, and medication and dietary supplement use. Each subject was informed of all procedures, potential risks, and benefits associated with the study in both verbal and written form in accordance with the approved procedures of the University Institutional Review Board for Human Subjects Research (H11-14). Subjects provided written informed consent prior to being enrolled in the study.

Subjects' height was measured using a stadiometer, and their bodyweight was measured using a calibrated scale. Body mass index was calculated as bodyweight (kg) divided by height squared (m^2^). Waist and hip circumferences were measured using a tension-regulated tape. Body composition was determined via dual energy x-ray absorptiometry (Hologic QDR-4500 W) using a 4-minute fan array. Subject characteristics are provided in Table 1.

**Table 1:** Descriptive data of men and women before and after a 21-day Daniel Fast

Variable	Pre	Post	p value
Bodyweight (kg)	77.8 ± 3.8	75.1 ± 3.5	0.60

Body mass index (kg·m^-2^)	26.1 ± 1.0	25.2 ± 0.9	0.52

Waist (cm)	88.7 ± 2.9	87.5 ± 2.9	0.77

Hip (cm)	101.3 ± 2.6	100.7 ± 2.4	0.85

Waist:Hip	0.88 ± 0.02	0.87 ± 0.02	0.76

Total Body Fat (%)	26.7 ± 2.0	26.1 ± 2.1	0.84

Trunk Body Fat (%)	26.7 ± 2.3	25.5 ± 2.4	0.72

Fat Mass (kg)	21.1 ± 2.2	19.9 ± 2.1	0.70

Fat Free Mass (kg)	56.7 ± 2.8	55.2 ± 2.7	0.70

Total Cholesterol (mg·dL^−1^)	178.0 ± 6.7	138.7 ± 5.5	<0.01

LDL cholesterol mg·dL^−1^)	107.4 ± 5.7	77.6 ± 4.9	<0.01

HDL Cholesterol mg·dL^−1^)	51.0 ± 2.5	45.9 ± 2.6	0.17

Data are mean±SEM.

Subjects were required to purchase and prepare all of their food during the 21-day fast.

Therefore, one purpose of the subjects' initial lab visit was to inform them of which foods they were and were not allowed to consume. An information packet was given to subjects that outlined which commonly consumed foods were and were not permissible to consume during the Daniel Fast. Subjects were also instructed to check the ingredients label of any packaged foods that they planned to consume for any ingredients that might disqualify the food from being Daniel Fast compliant (e.g., preservatives, additives, sweeteners, flavorings, caffeine, and alcohol). It should be noted that foods were not required to be organic; hence, some degree of pesticide use may have been present in the process of growing the foods consumed on this plan. Subjects were provided with food logs for dietary recording and given instructions on how to record their dietary intake. A basic recipe guide was also provided.

Within two weeks of the initial visit, subjects returned to the lab for the first test meal. After partaking in the Daniel Fast for 21 days, subjects returned to the lab for the second test meal. All data collection occurred in the morning hours (5:00-11:00 am). Subjects were instructed not to perform strenuous physical exercise during the 48 h before each test meal, as such activity may have impacted our outcome measures [24].

### Test days

Subjects reported to the lab on day one and day 22 to consume the test meals. On both occasions, subjects reported in a rested and 12-hour fasted state. Upon arrival to the lab, subjects were asked to void. Subjects were then seated in a chair with a blood pressure (BP) cuff placed on their left arm. After a 10-minute quiet rest period, two technicians measured heart rate (HR) by palpating the radial artery for 60 s. Blood pressure was then measured via auscultation using a dual-earpiece stethoscope that allowed for two technicians to listen simultaneously. Duplicate measures were obtained for both HR and BP, and the average of all measures was used in data analysis. If values deviated by more than 5 beats per minute for HR or 5 mm Hg for BP, an additional measure was taken. As an indicator of myocardial work, rate pressure product (RPP) was calculated as follows: HR × systolic BP. The rationale for measuring HR and BP was based on the observation that a postprandial rise in serum TAG is associated with an increase in ROS production [25], which may induce an acute state of endothelial dysfunction [26]. This in turn may lead to vascular dysfunction and elevated blood pressure during the postprandial period. Following measurement of HR and BP, a blood sample was obtained. The above procedures were repeated at 2 and 4 h following the ingestion of the milkshake (as described below).

### Test meal

The test meal was a milkshake that consisted of a combination of lipid, carbohydrate, and protein. Although it has been noted that lipid-only meals produce a more robust increase in circulating oxidative stress biomarkers than do mixed meals [21], such a pure fat load does not represent typical dietary intake. Therefore, for the sake of applicability, we used a lipid-rich, albeit mixed-macronutrient, milkshake; with a composition that is similar to milkshakes sold in many commercial establishments. Specifically, the milkshake was made using a combination of whole milk, ice cream (Breyers^® ^"all natural" vanilla), and heavy whipping cream. The milkshake's size (i.e., dietary energy content) was determined based on subjects' body mass; and equal to 0.8 g of fat, 1.0 g of carbohydrate, and 0.25 g of protein per kilogram body mass, totaling approximately 12.2 kcal per kilogram of body mass. This is a similar amount of lipid used in a prior study of postprandial oxidative stress [20] and less than that used in some of our prior work [22,23,27].

### Blood collection and biochemistry

Venous blood samples were taken from the subjects' antecubital vein via needle and Vacutainer™ before intake of the test meals and at 2 and 4 h following intake. We have noted in our prior work involving healthy adults that the peak TAG response to a high-fat milkshake occurs at two hours post ingestion, with oxidative stress biomarkers peaking between 2 and 4 h post ingestion. Blood samples that were collected in tubes containing EDTA were immediately separated via centrifugation at 1,500 g for 15 min at 4°C for collection of plasma. Blood samples that were collected in tubes containing no additives were allowed to clot at room temperature for 30 min and then separated by centrifugation at 1,500 g for 15 min at 4°C for collection of serum. Blood samples were immediately stored in multiple aliquots at −70°C until analyzed for the following variables:

Triglycerides (TAG) were analyzed in serum following standard enzymatic procedures as described by the reagent manufacturer (Thermo Electron Clinical Chemistry). Malondialdehyde (MDA) was analyzed in plasma following the procedures of Jentzsch et al. [28] using reagents purchased from Northwest Life Science Specialties (Vancouver, WA). Hydrogen peroxide (H_2_O_2_) was analyzed in plasma using the Amplex Red reagent method as described by the manufacturer (Molecular Probes, Invitrogen Detection Technologies, Eugene, OR). Advanced oxidation protein products (AOPP) were analyzed in plasma as previously described [29], with values presented as chloramine-t equivalents. Plasma NOx was analyzed using a commercially available colorimetric assay kit (Cayman Chemical, Ann Arbor, MI) according to the procedures provided by the manufacturer. Trolox Equivalent Antioxidant Capacity (TEAC) was analyzed in serum according to the procedures outlined by the reagent provider (Sigma Chemical, St. Louis, MO). Assays were performed in duplicate on first thaw.

### Dietary records, compliance, and physical activity

All subjects were instructed to maintain their normal diet until they began the fast. Subjects recorded all food and drink consumed during the 7 days prior to beginning the fast and the final 7 days of the fast. Diet records were reviewed with each subject for accuracy and then analyzed using Food Processor SQL (version 9.9; ESHA Research, Salem, OR). While attempts were made to accurately document food and beverage intake of our subjects (weekly contact via phone and email), we admit that self-reporting of dietary data can be met with problems--including under reporting and inaccurate reporting. This is indeed a limitation of this work. At the conclusion of the fast (day 22), subjects rated their compliance to the fast using a scale of 0-100 (0 = complete noncompliance; 100 = complete compliance). Subjects were instructed to maintain their normal physical activity and exercise habits during the entire study period with the following exception: Subjects were instructed not to perform strenuous exercise during the 48 h immediately preceding the two test meal days.

### Statistical analysis

Hemodynamic and biochemical measures were analyzed using a 2 (pre/post fast) × 3 (time) repeated measures analysis of variance (RMANOVA). Significant effects were further analyzed using Tukey's post hoc tests. For each biochemical measure, the area under the curve (AUC) was calculated using the trapezoidal method as described in detail by Pruessner et al. [30]. Descriptive and dietary variables were analyzed using a one-way ANOVA. Pairwise correlations were made between all biochemical variables. All analyses were performed using JMP statistical software (version 4.0.3, SAS Institute, Cary, NC). Statistical significance was set at *p *≤ 0.05. The data are presented as mean ± SEM.

## Results

### Compliance and dietary data

Subjects' self-reported compliance to the fast was 97.7 ± 0.8%. As expected, intake of several dietary variables changed from pre to post fast (*p *≤ 0.05), including total dietary energy. These changes in dietary intake are believed to be largely responsible for our observed effects. Dietary data are presented in Table 2.

**Table 2:** Dietary data of men and women before and during the final 7 days of a 21-day Daniel Fast

Variable	Pre	During	p value
Kilocalories	2046.3 ± 121.4	1719.1 ± 106.5	0.05

Protein (g)	94.2 ± 8.5	58.3 ± 4.9	<0.01

Carbohydrate (g)	249.8 ± 17.6	259.5 ± 19.3	0.71

Fiber (g)	23.3 ± 2.3	43.3 ± 3.6	<0.01

Sugar (g)	85.5 ± 7.3	75.6 ± 7.7	0.36

Fat (g)	71.5 ± 4.9	57.6 ± 5.2	0.06

Saturated Fat (g)	23.2 ± 1.7	8.9 ± 1.1	<0.01

Monounsaturated Fat (g)	14.3 ± 1.9	19.9 ± 3.3	0.15

Polyunsaturated Fat (g)	7.1 ± 0.7	9.1 ± 0.9	0.07

Trans Fat (g)	0.9 ± 0.2	0.3 ± 0.1	0.05

Omega-3 (mg)	0.6 ± 0.1	0.5 ± 0.1	0.12

Omega-6 (mg)	5.4 ± 0.6	5.8 ± 0.7	0.65

Cholesterol (mg)	266.6 ± 29.3	9.2 ± 5.7	<0.01

Vitamin C (mg)	75.3 ± 8.2	150.9 ± 17.4	<0.01

Vitamin E (mg)	7.3 ± 1.2	8.7 ± 1.1	0.40

Vitamin A (RE)	432.6 ± 62.9	520.1 ± 70.0	0.36

Selenium (μg)	57.4 ± 6.4	49.7 ± 15.6	0.65

Data are mean±SEM.

### Hemodynamic variables

**Figure 1:**
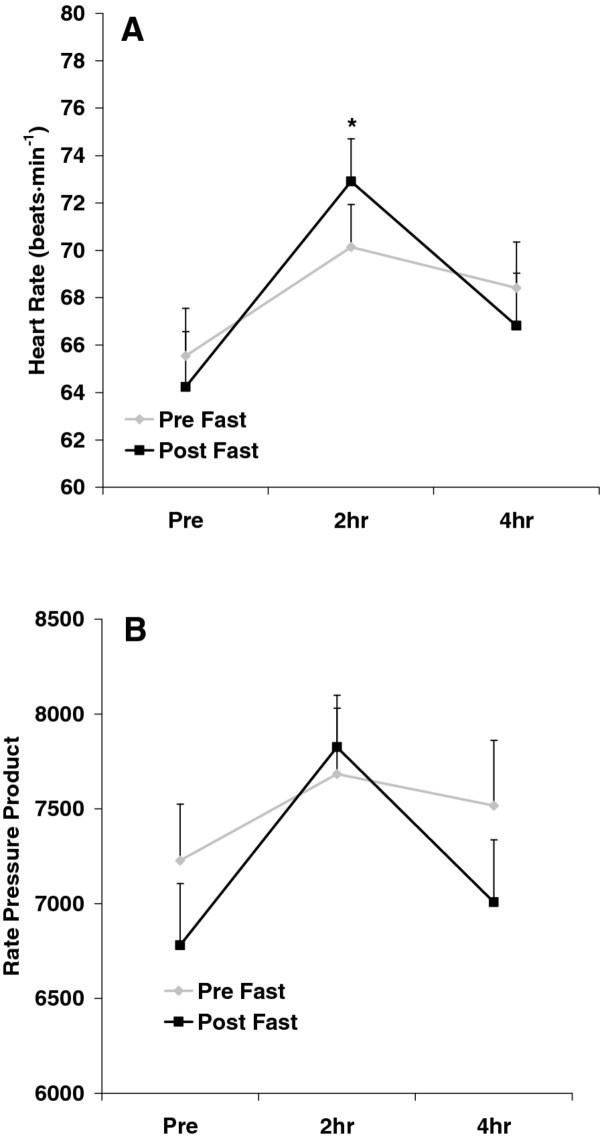
**Heart rate (A) and rate pressure product (B) before and after intake of a high-fat milkshake, before and after a 21-day Daniel Fast**. Values are mean±SEM. * Time effect for HR (*p *= 0.006); values higher than Pre at 2 h (*p *< 0.05). No other significant effects noted (*p *> 0.05).

**Figure 2:**
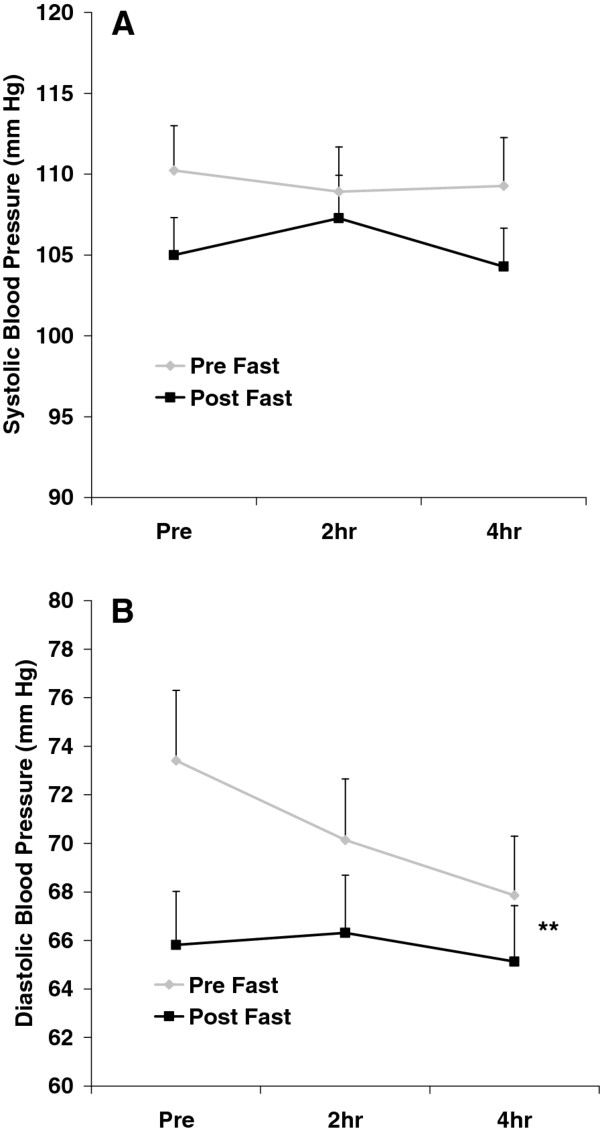
**Systolic (A) and diastolic (B) blood pressure before and after intake of a high-fat milkshake, before and after a 21-day Daniel Fast**. Values are mean±SEM. ** Pre/Post effect for DBP (*p *= 0.02). No other significant effects noted (*p *> 0.05).

No pre/post fast × time interactions were noted for any hemodynamic variable (*p *> 0.05). A time effect was noted for HR (*p *= 0.006), with values higher at 2 h post intake of the milkshake compared to pre intake (*p *< 0.05). Diastolic blood pressure was lower post fast as compared to pre fast (*p *= 0.02). Systolic blood pressure was also lower post fast as compared to pre fast, but this change did not reach statistical significance (*p *= 0.07). No other significant effects were noted for any hemodynamic variable (*p *> 0.05). Heart rate and RPP data are presented in Figure 1. Blood pressure data are presented in Figure 2.

### Biochemical variables

**Figure 3:**
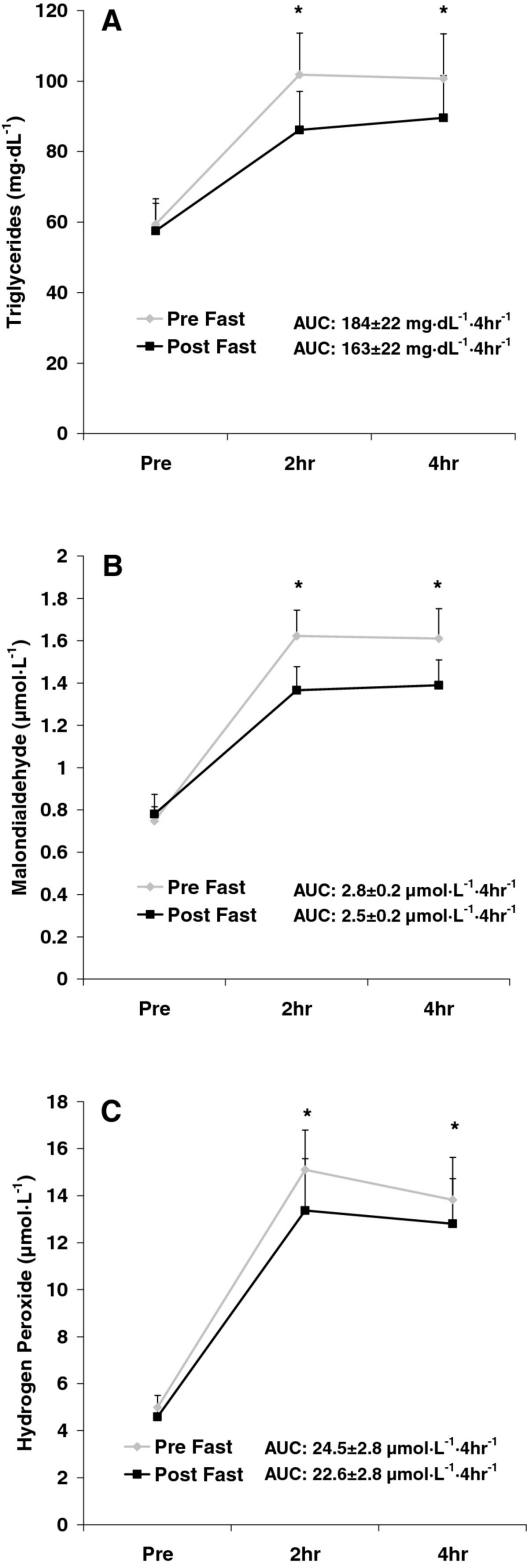
**Blood triglycerides (A), malondialdehyde (B), and hydrogen peroxide (C) before and after intake of a high-fat milkshake, before and after a 21-day Daniel Fast**. Values are mean±SEM. * Time effect for TAG (*p *= 0.001), MDA (*p *< 0.0001), and H_2_O_2 _(*p *< 0.0001); values higher than Pre at 2 h and 4 h (*p *< 0.05). No other significant effects noted (*p *> 0.05).

**Figure 4:**
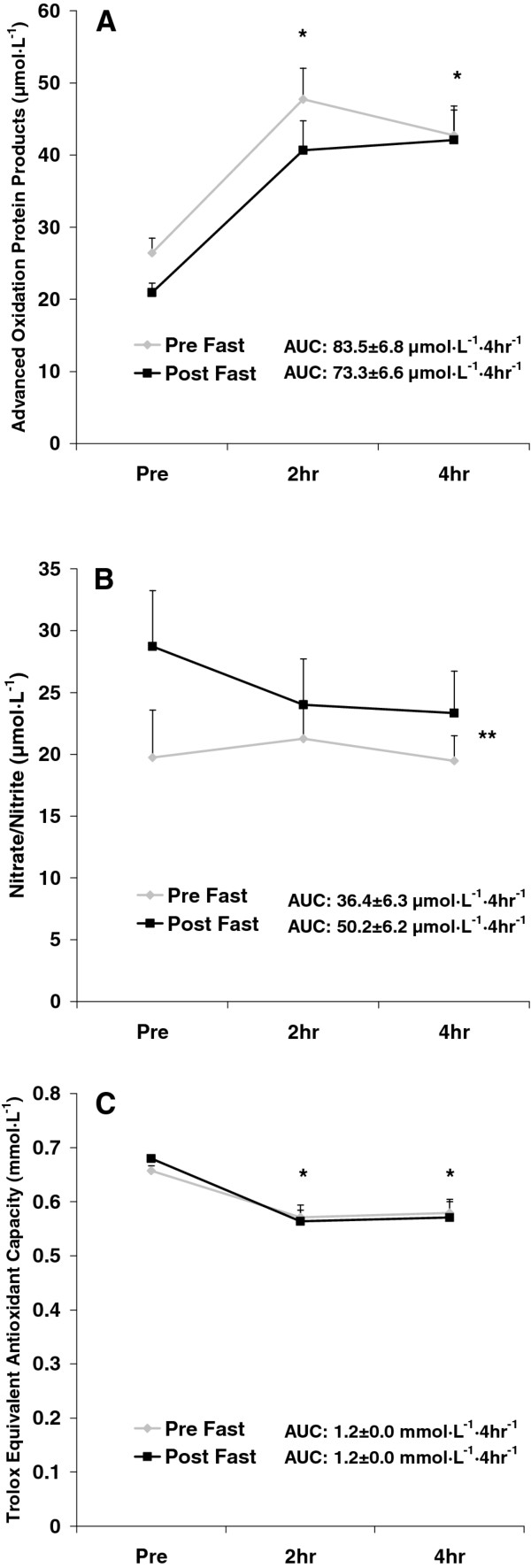
**Blood advanced oxidation protein products (A), nitrate/nitrite (B), and Trolox Equivalent Antioxidant Capacity (C) before and after intake of a high-fat milkshake, before and after a 21-day Daniel Fast**. Values are mean±SEM. * Time effect for AOPP (*p *< 0.0001); values higher than Pre at 2 h and 4 h (*p *< 0.05). * Time effect for TEAC (p < 0.0001); values lower than Pre at 2 h and 4 h (*p *< 0.05). ** Pre/Post effect for NOx (*p *= 0.02); values higher post fast compared to pre fast No other significant effects noted (*p *> 0.05).

No pre/post fast x time interactions were noted for any biochemical variable (*p *> 0.05). Area under the curve values from pre to post fast were not statistically different for TAG (*p *= 0.50), MDA (*p *= 0.24), H_2_O_2 _(*p *= 0.63), AOPP (*p *= 0.29), NOx (*p *= 0.12), or TEAC (*p *= 0.99). While not statistically significant, the AUC was slightly lower from pre to post fast for TAG (11%), MDA (11%), H_2_O_2 _(8%), and AOPP (12%); and a 37% increase in the AUC for NOx was also noted. No change was noted in the AUC for TEAC. Time effects were noted for TAG (*p *= 0.001), MDA (*p *< 0.0001), H_2_O_2 _(*p *< 0.0001), AOPP (*p *< 0.0001), and TEAC (*p *< 0.0001); all values were higher at 2 h and 4 h post intake compared to pre intake, except for TEAC, which was lower at 2 h and 4 h post intake compared to pre intake (*p *< 0.05). A condition effect was noted for NOx (*p *= 0.02), which was higher post fast as compared to pre fast. No other significant effects were noted for any biochemical variable (*p *> 0.05). Data for TAG, MDA, and H_2_O_2 _are presented in Figure 3. Data for AOPP, NOx, and TEAC are presented in Figure 4. Significant (*p *≤ 0.01) and strong correlations were noted between all biochemical variables except for TEAC and NOx (*p *= 0.39), as indicated in Table 3.

**Table 3:** Correlation matrix for biochemical variables

	TAG	MDA	H_2_O_2_	AOPP	NOx	TEAC
TAG	1.0	0.84	0.71	0.74	0.21	-0.54

MDA	0.84	1.0	0.80	0.81	0.22	-0.59

H_2_O_2_	0.71	0.80	1.0	0.88	0.25	-0.58

AOPP	0.74	0.81	0.88	1.0	0.25	-0.66

NOx	0.21	0.22	0.25	0.25	1.0	-0.08*

TEAC	-0.54	-0.59	-0.58	-0.66	-0.08*	1.0

Values are correlation coefficients.

*p = 0.39; All other correlation coefficients are significant (*p *≤ 0.01)

*TAG *triglycerides; *MDA *malondialdehyde; *H_2_O_2 _*hydrogen peroxide; *AOPP *advanced oxidation protein products; *NOx *nitrate/nitrite; *TEAC *trolox equivalent antioxidant capacity

## Discussion

Findings from the present investigation indicate that modifying dietary intake in accordance with a 21-day Daniel Fast 1) does not impact the hemodynamic or biochemical response to a high-fat meal, 2) lowers fasting BP, and 3) increases NOx. As it is currently unknown what specific impact these outcomes have on health, longer-term studies are needed to explore this line of inquiry.

Partaking in the Daniel Fast did not affect the hemodynamic response to the test meal. This may have been due to the fact that the test meal by itself had little effect on the hemodynamic variables measured in this study, thus leaving little room for improvement. However, fasting did lower pre-meal systolic and diastolic BP by 5 mm Hg and 8 mm Hg, respectively (Figure 2), despite the fact that subjects' pre-fast BP was low and within normal range.

With reference to antioxidant capacity, we failed to note an increase in TEAC from pre to post fast, which opposes the results of our initial Daniel Fast study [12]. In fact, average TEAC values in the present study were nearly identical at all times of measurement (Figure 4). The lack of increase in TEAC may be explained in at least two ways: First, it is likely that the TEAC measure does not capture in a sensitive manner all changes in the antioxidant pool (e.g., phenol antioxidants, thiols, enzymes), as TEAC is simply a measure of "global" antioxidant capacity. Second, despite the potential increase in dietary antioxidant intake in the form of whole foods from pre to post fast (some of which may have contributed to TEAC and some of which may have not), the omission of coffee from subjects' diet during the fast may have resulted in a lower TEAC value (Increased whole-food antioxidants may have counteracted this decrease to allow for a *maintenance *in TEAC.). This is because coffee has been reported to contribute to the majority of daily antioxidant intake [31]. Although the Daniel Fast prohibits coffee consumption when partaken as a spiritual discipline, studies examining the Daniel Fast from a purely clinical perspective may consider allowing subjects who regularly consume coffee to continue doing so in an attempt to maintain their dietary antioxidant intake.

Along with the absence of change in TEAC from pre to post fast, we noted near identical concentrations for pre-meal TAG (Figure 3). In our prior work with the Daniel Fast, we noted modest reductions in fasting TAG (11.5%), which we hypothesized might influence postprandial TAG concentrations, as such an effect has been reported previously [22]. Neither the pre-meal or post meal TAG concentrations were different from pre to post fast. In much the same way as discussed for TEAC, it is apparent that pre-meal TAG concentrations do not completely influence the postprandial oxidative stress response to feeding. It is possible that other blood lipids such as cholesterol play a role in this response, as both total and LDL cholesterol concentrations decreased from pre to post fast (Table 1). The fact that we did not measure the postprandial cholesterol response is a limitation of this work. Furthermore, a decrease in the production of ROS from pre to post fast likely plays a significant role in the resulting lowering in oxidative stress biomarkers. Direct assessment of ROS using electron spin resonance spectroscopy was not performed in the present design, which may be considered a limitation of this work.

In relation to ROS production, our noted decrease in total dietary energy, in addition to dietary protein (Table 2), was thought to decrease ROS and the oxidative stress response to feeding, as both dietary energy [9] and protein restriction [10,11] is associated with decreased ROS. However, despite a decrease in kilocalorie intake of approximately 16% and protein intake of approximately 38%, we observed no statistically significant reduction in postprandial oxidative stress. While a greater reduction in these dietary parameters (or a longer time course of dietary intervention) may lead to more robust decreases in our chosen oxidative stress biomarkers, we must question the benefit of such further reduction. That is, participants consumed a relatively low kilocalorie diet and consumed an average of only 58.3 ± 4.9 g of protein daily during the Daniel Fast, which is slightly less than the current recommended amount of 0.8 g·kg^-1^. Any further reductions may not be well-tolerated. Rather, maintaining this degree of reduction for a longer time period may be necessary and met with more favorable results.

Our failure to note findings of statistical significance may be partly due to our relatively small sample size. Moreover, although we included a variety of oxidative stress biomarkers in our design, additional measures may be considered in future experiments to better capture the redox status during the postprandial period. Of course, the physiological implications of any potential biochemical change need to be explored in future work with the inclusion of clinical measures (e.g., endothelial function, future cardiovascular disease risk). While elevated oxidative stress [32] and impaired nitric oxide production [33] appear to be correlated with disease progression, it is unknown what impact small changes in these variables might have on healthy individuals. This is emphasized when considering the relatively low values (both pre and post fast) that our subjects demonstrated for the measured variables (e.g., pre fast TAG values of 59 ± 7 mg dL^-1^). Such low values in our sample of healthy, active subjects may have impaired our ability to note decreases of statistical significance from pre to post fast. The inclusion of subjects with hyperlipidemia may have allowed for greater potential for change in our measured variables from pre to post fast.

As expected, TAG, MDA, H_2_O_2_, AOPP, and TEAC were each affected by the test meal consumption. Each of these values (except for TEAC) increased at 2 and 4 h post meal compared to pre meal, while TEAC decreased at these times. These findings replicate our prior work using high-fat meals to induce oxidative stress [20,21]. In addition, NOx increased from pre to post fast, which may have implications with regards to vascular health and enhanced blood flow [33,34].

While we failed to note any change of statistical significance in postprandial oxidative stress biomarkers from pre to post fast, subjects did appear to process the test meal more efficiently after the fast as compared to before the fast (as evidenced by an approximate 10% reduction in TAG and oxidative stress biomarkers). While this was our working hypothesis, we acknowledge the possibility that reducing the consumption of saturated fat and eliminating the consumption of processed foods for 21 days may have made subjects *more *susceptible to the harmful effects of a high- fat meal. In much the same way as a "repeated bout effect" has been noted for exercise [35], it is possible that such an effect may be present with routine high-fat feeding. That is, regular intake of high-fat meals may not present as significant of a metabolic stress due to the fact that the body is familiar with such a stressor and has adapted to handle this accordingly. Although this explanation was not the case in the present study, as subjects responded in a similar (or slightly more favorable) manner to the high-fat test meal despite abstaining from such foods for a 21-day period, such an effect may have negated some of the benefits of the Daniel Fast, resulting in our non-significant findings.

Although not a primary interest in the present design, we computed the correlations between each of our biochemical variables, including TAG. Table 3 provides these data, which corroborate our prior work demonstrating both significant and strong correlations between blood TAG and a variety of oxidative stress biomarkers [20-23]. As TAG is routinely measured in a clinical setting, while biomarkers of oxidative stress are not, it seems plausible that the simple measurement of serum TAG following administration of an oral fat tolerance test could serve as an estimation of postprandial oxidative stress. Future work using a larger data set inclusive of both TAG and oxidative stress biomarkers is needed in order to generate the requisite prediction equations. Furthermore, as the TAG response to feeding is strongly correlated to all measures of oxidative stress, it seems logical that attenuation in postprandial TAG should be the first line defense against an increase in postprandial oxidative stress. In fact, blunting the TAG response to high-fat feeding may be much more important than increasing antioxidant defense. In this way, the problem (ROS production) is controlled *before *it presents itself and subsequently needs to be dealt with (via increased antioxidant defense). Interventions (both chronic and acute) aimed at minimizing the TAG response to feeding should be investigated (as has been done successfully with exercise [36]), with a particular focus on understanding the molecular mechanisms relating elevated TAG and increased oxidative stress and disease.

## Conclusions

Partaking in a 21-day Daniel Fast does not impact the biochemical response to a high-fat meal. Controlling the TAG response to feeding may be most important with regards to attenuating postprandial oxidative stress. Individuals with known cardiovascular or metabolic disease, particularly hyperlipidemic individuals who do not currently consume a "healthy" diet, may be needed to demonstrate more favorable effects in our chosen outcome measures. A longer postprandial observation period may be considered in future research in an attempt to more fully elucidate the time course of response in oxidative stress biomarkers. Finally, future investigations may consider incorporating fasting periods of longer than 21 days in order to determine success with regards to both compliance and outcome measures.

## Competing interests

The authors declare that they have no competing interests.

## Authors' contributions

RJB was responsible for the study design, performance of biochemical assays, statistical analyses, and writing of the manuscript. JFT and MMK were responsible for coordination of the study. JFT, MMK, and RJA were responsible for data collection and entry. MED was responsible for performing the AOPP assays. All authors assisted in reviewing/editing the manuscript and all authors approved of the final manuscript.
